# Correction: Interventions for treating children and adolescents with overweight and obesity: an overview of Cochrane reviews

**DOI:** 10.1038/s41366-019-0358-4

**Published:** 2019-04-02

**Authors:** Louisa J. Ells, Karen Rees, Tamara Brown, Emma Mead, Lena Al-Khudairy, Liane Azevedo, Grant J. McGeechan, Louise Baur, Emma Loveman, Heather Clements, Pura Rayco-Solon, Nathalie Farpour-Lambert, Alessandro Demaio

**Affiliations:** 1 School of Health and Social Care, Teesside University, Middlesbrough, UK; 2 Division of Health Sciences, University of Warwick, Coventry, UK; 3 University of Nottingham, Nottingham, UK; 4 University of Sydney, Sydney, NSW Australia; 5 Effective Evidence LLP, Hampshire, UK; 6 World Health Organization, Geneva, Switzerland; 7 Obesity Prevention and Care Program, University Hospitals of Geneva, Geneva, Switzerland

**Correction to: International Journal of Obesity** **42**, 1823–1833;

10.1038/s41366-018-0230-y;

published online 09 October 2018

This paper was originally published under a standard licence. This has now been amended to a CC BY licence in the PDF and HTML.

